# Increased PRL-1 in BM-derived MSCs triggers anaerobic metabolism via mitochondria in a cholestatic rat model

**DOI:** 10.1016/j.omtn.2023.01.017

**Published:** 2023-02-04

**Authors:** Jae Yeon Kim, Se Ho Kim, Jin Seok, Si Hyun Bae, Seong-Gyu Hwang, Gi Jin Kim

**Affiliations:** 1 Department of Biomedical Science, CHA University, 689, Sampyeong-dong, Bundang-gu, Seongnam-si 13488, Republic of Korea; 2 Research Institute of Placental Science, CHA University, Seongnam 13488, Republic of Korea; 3 Department of Internal Medicine, Catholic University Medical College, Seoul 03312, Republic of Korea; 4 Department of Gastroenterology, CHA Bundang Medical Center, CHA University School of Medicine, Seongnam 13496, Republic of Korea

**Keywords:** MT: Delivery strategies, BM-MSCs, cholestasis, mitochondria, PRL-1, stem cell therapy

## Abstract

Mesenchymal stem cell (MSC) therapy in chronic liver disease is associated with mitochondrial anaerobic metabolism. Phosphatase of regenerating liver-1 (*PRL-1*), known as protein tyrosine phosphatase type 4A, member 1 (*PTP4A1*), plays a critical role in liver regeneration. However, its therapeutic mechanism remains obscure. The aim of this study was to establish genetically modified bone marrow (BM)-MSCs overexpressing PRL-1 (BM-MSCs^PRL−1^) and to investigate their therapeutic effects on mitochondrial anaerobic metabolism in a bile duct ligation (BDL)-injured cholestatic rat model. BM-MSCs^PRL−1^ were generated with lentiviral and nonviral gene delivery systems and characterized. Compared with naive cells, BM-MSCs^PRL−1^ showed an improved antioxidant capacity and mitochondrial dynamics and decreased cellular senescence. In particular, mitochondrial respiration in BM-MSCs^PRL−1^ generated using the nonviral system was significantly increased as well as mtDNA copy number and total ATP production. Moreover, transplantation of BM-MSCs^PRL−1^ generated using the nonviral system had predominantly antifibrotic effects and restored hepatic function in a BDL rat model. Decreased cytoplasmic lactate and increased mitochondrial lactate upon the administration of BM-MSCs^PRL−1^ indicated significant alterations in mtDNA copy number and ATP production, activating anaerobic metabolism. In conclusion, BM-MSCs^PRL−1^ generated by a nonviral gene delivery system enhanced anaerobic mitochondrial metabolism in a cholestatic rat model, improving hepatic function.

## Graphical abstract

**Figure:**
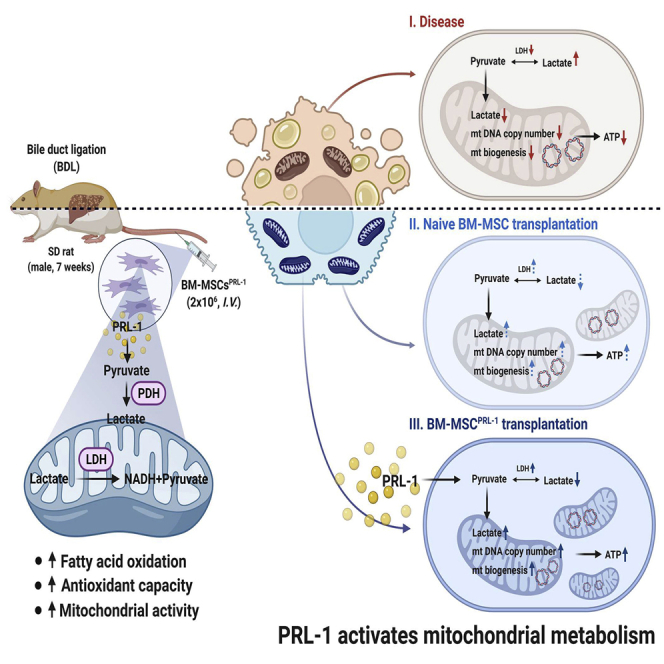

## Teaser

We successfully generated BM-MSCs^PRL−1^ using gene delivery systems. It improved antioxidant capacity via mitochondrial dynamics and decreased cellular senescence in injured hepatocytes. It had antifibrotic effects by enhancing anaerobic metabolism. BM-MSCs^PRL−1^ generated by a nonviral gene delivery system enhanced anaerobic mitochondrial metabolism in a cholestatic rat model, improving hepatic function.

## Introduction

Chronic liver disease is a major hepatic disorder with metabolic syndrome^1^ that encompasses simple liver fat accumulation to more progressive steatosis, inflammation, and fibrosis and, in some cases, cirrhosis and hepatocellular carcinoma.^2^^,^^3^ Metabolic syndrome is also associated with worse outcomes in patients with cirrhosis due to hypometabolism and anaerobic glycolysis.^4^^,^^5^ In particular, cholestatic liver diseases involve a reduction in bile flow and the accumulation of toxic bile acids in the liver and systemic circulation. These conditions can be induced by the inflammatory response, oxidative stress, increased apoptosis, and eventually liver fibrosis. Chronic cholestasis results in increased bile ductules, hepatomegaly, the appearance of regenerative nodules, decreased enterohepatic circulation of bile acids, and portal hypertension. Therefore, the main cause of cholestasis involves intense metabolic alterations such as changes in mitochondrial energy production.^6^ The inhibitory activities of the respiratory chain in cholestatic hepatocytes impair the synthesis of mitochondrial proteins, resulting in defective mitochondrial biogenesis. Moreover, mitochondrial respiration can fuel lactate production and the conversion of lactate to pyruvate.^7^ Mitochondrial lactate dehydrogenase (LDH) plays an important role in tissue lactate clearance and oxidation for ATP production.^8^ In intrinsic hepatic disease, in which the liver blood flow is reduced to 25% of normal, lactate clearance is reduced. With severe shock, lactate uptake by the monocarboxylate transporter becomes saturated, the development of intracellular acidosis inhibits gluconeogenesis, and reduced liver blood flow delivers less lactate for metabolism.^9^^,^^10^ Additionally, the accumulation of bile acids negatively affects mitochondrial bioenergetics by impairing respiration, increasing oxidative stress, and decreasing ATP synthesis. However, mitochondrial lactate metabolism in liver diseases is still unknown.

In chronic liver diseases, mesenchymal stem cell (MSC) transplantation for hepatic restoration involves several cellular dynamics based on a mitochondria-dependent pathway. Upon the administration of bone marrow-derived MSCs (BM-MSCs) in an acute liver injury model, the decreased apoptosis of hepatocytes was confirmed by increased *Bcl2* expression.^11^ In patients with liver cirrhosis, BM-MSC transplantation increased the number of mitochondria in hepatocytes with multiple cristae.^12^ Moreover, bone marrow mononuclear cell transplantation in bile duct ligation (BDL)-injured rats decreased mitochondrial oxidative stress and mitochondrial uncoupling, resulting in improved mitochondrial bioenergetics and liver function.^13^ Autologous BM-MSCs in patients with alcoholic cirrhosis have been examined in clinical trials.^14^ Several studies have shown that BM-MSC infusion can restore hepatic function.^15^^,^^16^ However, isolated autologous BM-MSCs from patients with cirrhosis had low qualities, such as a reduced mitochondrial membrane potential and decreased ATP levels.^17^ For this reason, the use of naive MSC-based cell therapy to target disease restricts the therapeutic mechanism and function of MSCs, as their self-renewal and senescence properties are limited, according to previous studies.

Phosphatase of regenerating liver-1 (*PRL-1*), also known as protein tyrosine phosphatase type 4A, member 1 (*PTP4A1*), was identified as an immediate-early gene during hepatic regeneration.^18^ In addition to *PRL-1*, *2*, and *3* are additional isoforms with similar amino acid sequences.^19^ PRLs have a catalytic domain that is similar to the dual-specificity protein tyrosine phosphatase domain.^20^ In particular, *PRL-1* showed potential in defending against oxidative stress levels by regulating upstream of the glutathione system^21^ and controls cellular energy metabolism via the activation of AMP-activated protein kinase (AMPK), which regulates magnesium homeostasis.^22^ However, the effect of *PRL-1* anaerobic metabolism on MSC properties remains unclear.

The objectives of the present study are to generate *PRL-1*-modified BM-MSCs (BM-MSCs^PRL−1^) using gene delivery systems and to analyze their therapeutic effects on mitochondrial anaerobic metabolism in a cholestatic rat liver model with BDL.

## Results

### Characterization of BM-MSCs^PRL−1^ generated using different gene delivery systems

**Figure 1:**
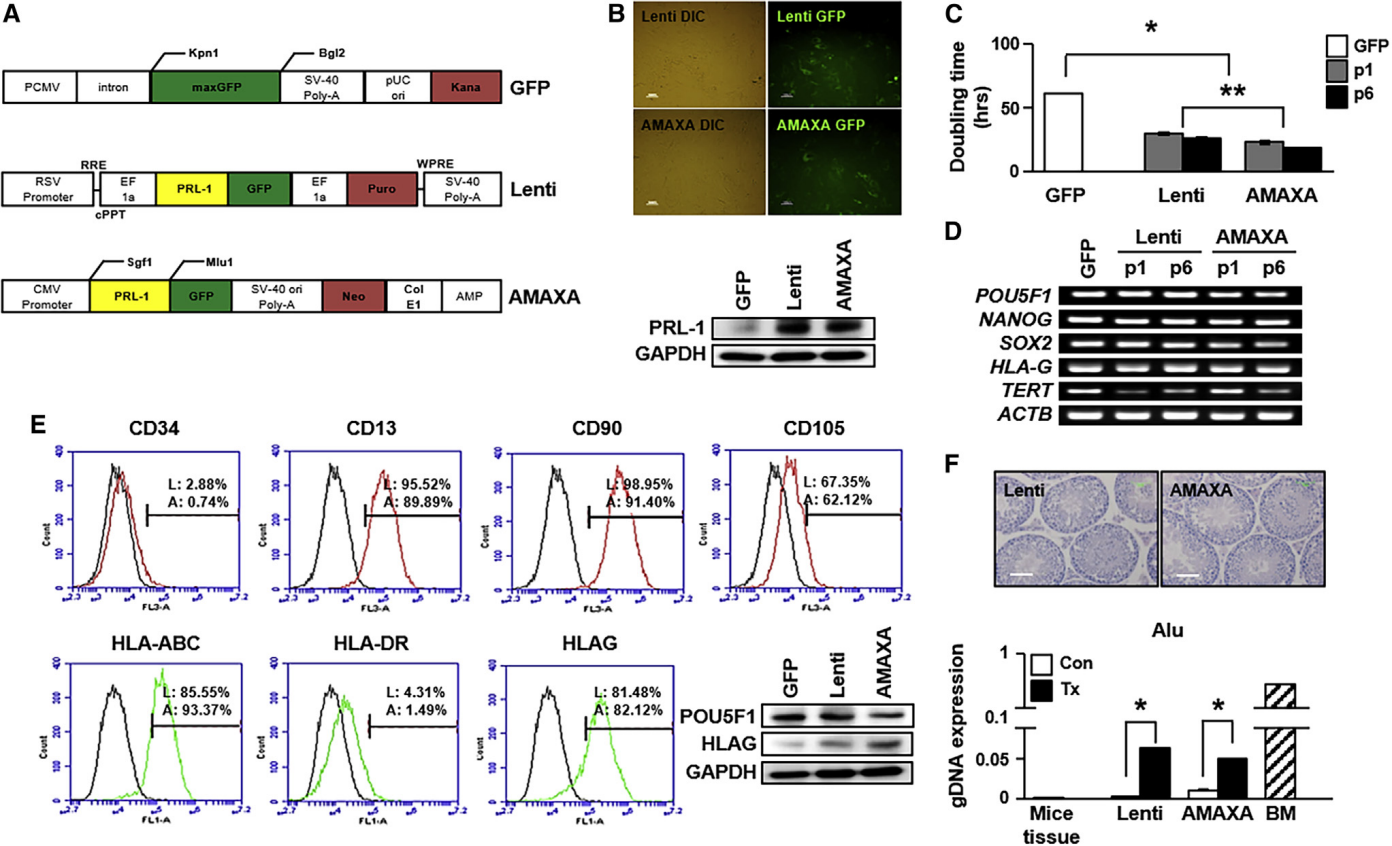
Characterization of BM-MSCs^PRL−1^ generated using gene delivery systems (A) GFP, lentiviral, and nonviral AMAXA plasmid vector maps. (B) GFP expression in BM-MSCs^PRL−1^ generated using the lentiviral and nonviral AMAXA systems, including bright field images. Scale bars, 100 μm. The expression of PRL-1 in BM-MSCs generated with each transfection system. (C) DT of BM-MSCs^PRL−1^ according to passage number. (D) RT-PCR analysis of stemness markers in naive BM-MSCs and BM-MSCs^PRL−1^ depending on passage number. ^∗^p < 0.05 in comparison with the GFP group, ∗∗p < 0.05 in comparison with the lenti group. (E) Fluorescence-activated cell sorting (FACS) analysis of surface markers related to hematopoietic cells and nonhematopoietic cells, and HLA family members in BM-MSCs^PRL−1^. Western blotting of POU5F1 and HLAG expression in naive BM-MSCs and BM-MSCs^PRL−1^. (F) H&E staining of normal and BM-MSC^PRL−1^-transplanted NOD/SCID mouse testes at 14 weeks (Lenti, n = 2; AMAXA, n = 2). Scale bar, 50 μm. Engraftment of BM-MSCs^PRL−1^ into mouse testes post transplantation. Mouse tissue was used as a negative control. Human BM-MSCs were used as a positive control. Values represent the mean ± SD. ∗p < 0.05 in comparison with the Con group.

BM-MSCs^PRL−1^ were generated with specific plasmids using either a lentiviral system or an electroporation-based nonviral P1 Primary Cell 4D Nucleofector X Kit L for Human MSCs (Lonza) as well as GFP (Figure 1A). After transfection, both types of *PRL-1*-overexpressing BM-MSCs expressed GFP, and the protein expression levels of *PRL-1* were increased in BM-MSCs^PRL−1^ compared with BM-MSCs^GFP^ (Figure 1B). Interestingly, the BM-MSC^PRL−1^ doubling time (DT) was significantly decreased compared with that of naive BM-MSCs^GFP^; otherwise, expression of the stemness markers POU Class 5 Homeobox 1 (*POU5F1*), *NANOG*, *SOX2*, and telomerase reverse transcriptase (*TERT*) and displayed human leukocyte antigen G (*HLAG*) were similar to those of naive BM-MSC^GFP^ (Figures 1C and 1D; p < 0.05). Additionally, upon immunophenotype analysis, the BM-MSCs^PRL−1^ generated using the lentiviral and AMAXA systems were positive for MSC markers (CD13, CD90, and CD105) and negative for hematopoietic stem cell markers (CD34). In addition, the presence of human leukocyte antigen (HLA)-ABC (MHC class I) and HLAG and absence of HLA-DR (MHC class II) were found in both naive BM-MSCs and BM-MSCs^PRL−1^. The protein expression levels of POU5F1 and HLAG in BM-MSCs^PRL−1^ were consistent with those in naive cells (Figures 1E and S1). To assess pluripotency *in vivo*, teratoma formation was induced by direct injection of BM-MSCs^PRL−1^ in the testes of 9-week-old male NOD/SCID mice (n = 2, lentiviral; n = 2, AMAXA). Fourteen weeks after injection, NOD/SCID mice were sacrificed, and each testis was evaluated by H&E staining. Testes injected with BM-MSCs^PRL−1^ generated using both systems and noninjected testes displayed no teratoma formation. Expression of the human-specific Alu sequence in the testis tissue was detected after BM-MSC^PRL−1^ injection (Figure 1F). In addition, these cells have multidifferentiation capacities, including mesodermal, endodermal, and ectodermal lineages (Figures S2 and S3). These findings show that BM-MSCs^PRL−1^ were successfully generated and characterized as being similar to BM-MSCs^GFP^.

### Antioxidant capacity of BM-MSCs^PRL−1^ through the regulation of cellular senescence

**Figure 2:**
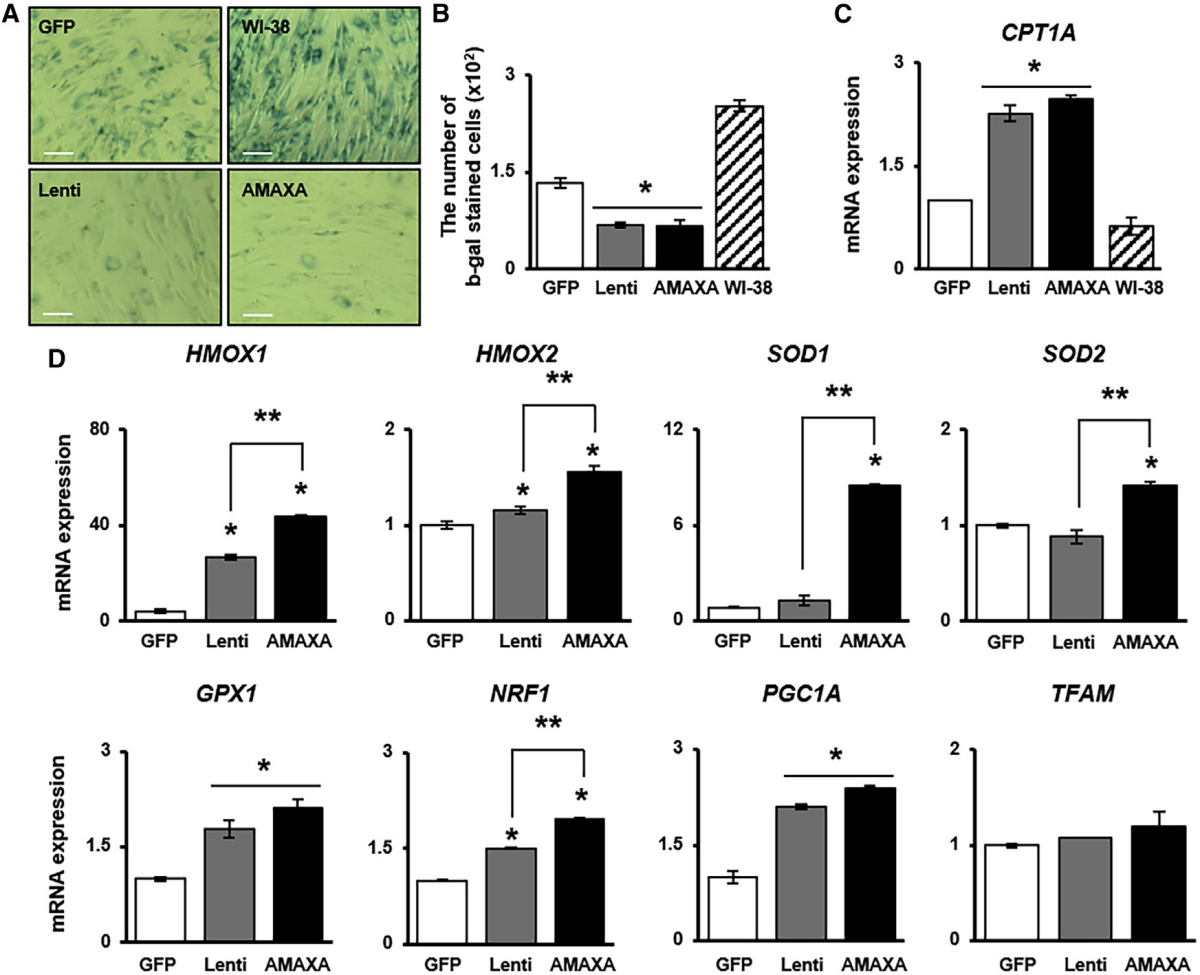
Antioxidant capacities of BM-MSCs^PRL−1^ generated using gene delivery systems through their regulation of cellular senescence (A) SA-β-gal staining of BM-MSCs^PRL−1^. WI-38 cells were used as positive control. Scale bars, 50 μm. (B) Number of SA-β-gal-positive BM-MSCs^PRL−1^. Scale bars, 100 μm. (C) qRT-PCR analysis of CPT1A, (D) antioxidant marker (e.g., *HMOX1*, *2*, *SOD1*, *2*, and *GPX1*) and mitochondrial biogenesis marker (e.g., *NRF1*, *PGC1A*, and *TFAM*) expression in BM-MSCs^PRL−1^. Values represent the mean ± SD. ∗p < 0.05 in comparison with the GFP group; ∗∗p < 0.05 in comparison with the lentiviral group.

To investigate whether *PRL-1* regulates redox signaling as a mediator of cellular senescence, BM-MSCs^PRL−1^ senescence and the gene expression of antioxidant enzymes were analyzed. Regardless of the gene delivery system, the proportion of senescence-associated beta-galactosidase (SA-β-gal)-positive cells among BM-MSCs^PRL−1^ was decreased compared with that among BM-MSCs^GFP^ (Figures 2A and 2B). Additionally, the mRNA expression of carnitine palmitoyltransferase 1 (*CPT1A*), which is the key factor in mitochondrial fatty acid β-oxidation, was increased in BM-MSCs^PRL−1^ compared with BM-MSCs^GFP^ (Figure 2C). Interestingly, BM-MSCs^PRL−1^ generated using the nonviral AMAXA system showed dramatically increased the mRNA levels of antioxidant enzymes (e.g., heme oxygenase; *HMOX 1*, *2*, superoxide dismutase; *SOD 1*, *2*, glutathione peroxidase 1; *GPX1*) compared with those upon the use of the lentiviral system. Additionally, mitochondrial biogenesis markers (e.g., nuclear respiratory factor 1 [*NRF1*], peroxisome proliferator activated receptor gamma coactivator 1 alpha [*PGC1A*], mitochondrial transcription factor A [*TFAM*]) were confirmed by qRT-PCR (Figure 2D; p < 0.05). These data suggest that BM-MSCs^PRL−1^ generated using different gene delivery systems have antioxidant effects and reduced cellular senescence.

### Effect of BM-MSCs^PRL−1^ on mitochondrial respiration

**Figure 3:**
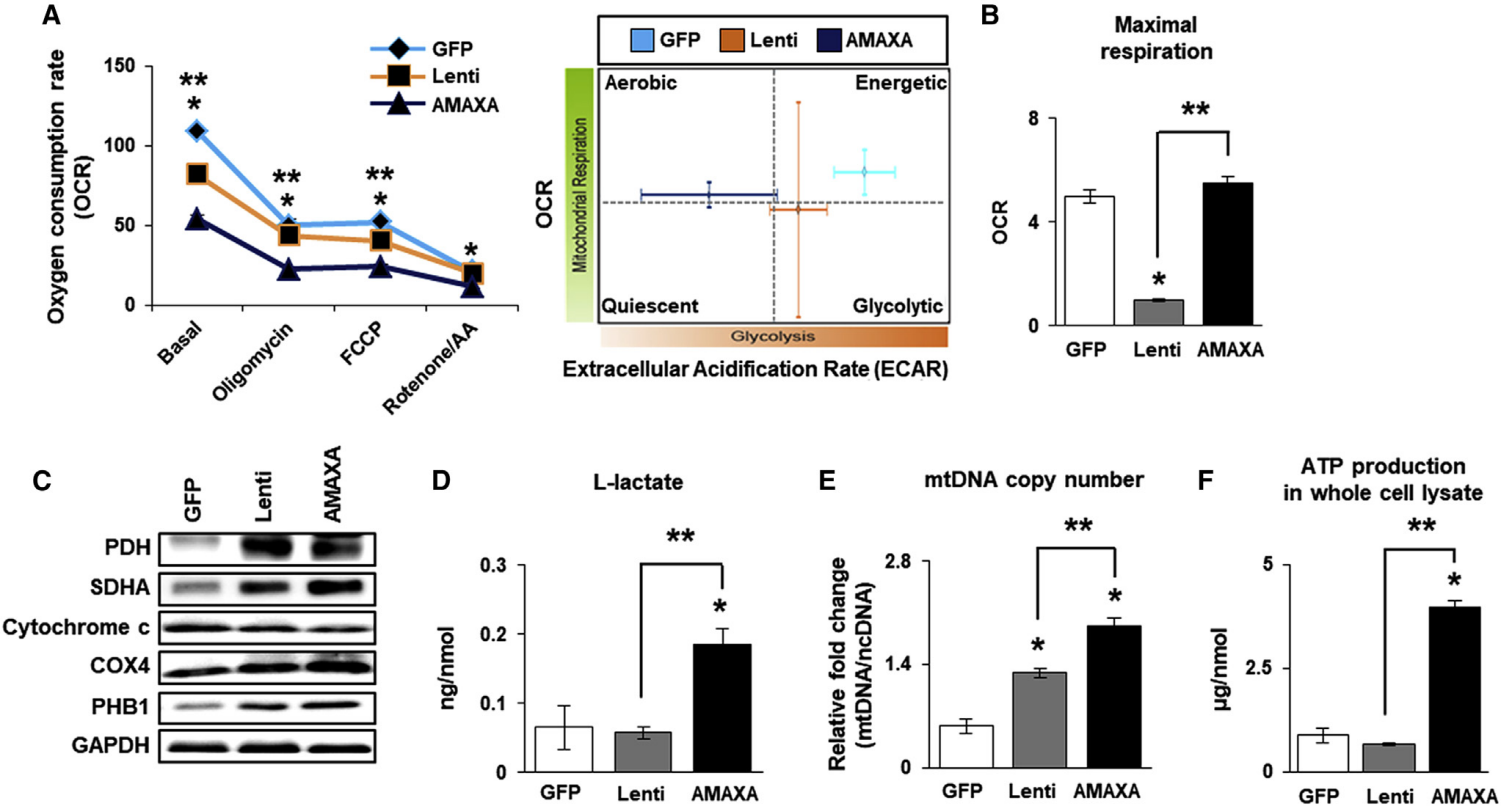
Mitochondrial respiration in BM-MSCs^PRL−1^ generated using gene delivery systems (A) OCR and ECAR of BM-MSCs^PRL−1^ generated using lentiviral and nonviral AMAXA systems after sequential treatment with 1 μM oligomycin, 0.5 μM FCCP, and 0.5 μM rotenone/AA determined with the Seahorse XF24 analyzer. (B) Quantification of the maximal respiration of BM-MSCs^PRL−1^ under live conditions. (C) Western blotting of mitochondrial metabolism-specific genes (e.g., PDH, SDHA, cytochrome *c*, COX4, and PHB1) in BM-MSCs^PRL−1^. (D) Assay of L-lactate production by BM-MSCs^PRL−1^ using a cell lysate (<25 mg/mL). (E) mtDNA copy number of BM-MSCs^PRL−1^ determined by the TaqMan assay. Human nuclear DNA was used as an internal control. (F) Assay of ATP production in BM-MSC^PRL−1^ lysate (10 μg/μL). Values represent the mean ± SD. ∗p < 0.05 in comparison with the GFP group. ∗∗p < 0.05 in comparison with the lentiviral group.

To further analyze mitochondrial respiration in BM-MSCs^PRL−1^, the XF Cell Mito Stress Test was performed to assess the effects of several mitochondrial complex inhibitors (e.g., oligomycin, carbonyl cyanide-4-(trifluoromethoxy) phenylhydrazone [FCCP] and rotenone/antimycin a [AA]) in the electron transport chain. Compared with BM-MSCs^PRL−1^ generated using either system, BM-MSCs^GFP^ displayed increased metabolism with regard to oxygen consumption rate (OCR) and extracellular acidification rate (ECAR). BM-MSCs^PRL−1^ generated using the lentiviral system showed a shift toward the more glycolytic state to maintain their energy balance, in contrast to the changes in aerobic state in BM-MSCs^PRL−1^ generated using the nonviral system (Figure 3A). However, the maximal respiration capacity, which may account for the capacity of cells to respond to an energy demand, of BM-MSCs^PRL−1^ generated using the nonviral system was increased compared with that of naive BM-MSCs and BM-MSCs^PRL−1^ generated using the lentiviral system (Figure 3B; p < 0.05). Although cytochrome *c* and cytochrome *c* oxidase (COX) 4 expression was no different, the protein expression of mitochondrial glycolic markers (e.g., pyruvate dehydrogenase [PDH], succinate dehydrogenase complex subunit A [SDHA], and prohibitin 1 [PHB1]) in BM-MSCs^PRL−1^ was higher than that in BM-MSCs^GFP^ (Figure 3C). Interestingly, lactate production at the cellular level, mtDNA copy number, and ATP production were different between lentiviral and AMAXA groups (Figures 3D–3F; p < 0.05). These data indicate that BM-MSCs^PRL−1^ generated using the nonviral system used mitochondria as their energy source and showed improved aerobic glycolysis.

### BM-MSCs^PRL−1^ had predominantly antifibrotic effects in a BDL-injured rat model

**Figure 4:**
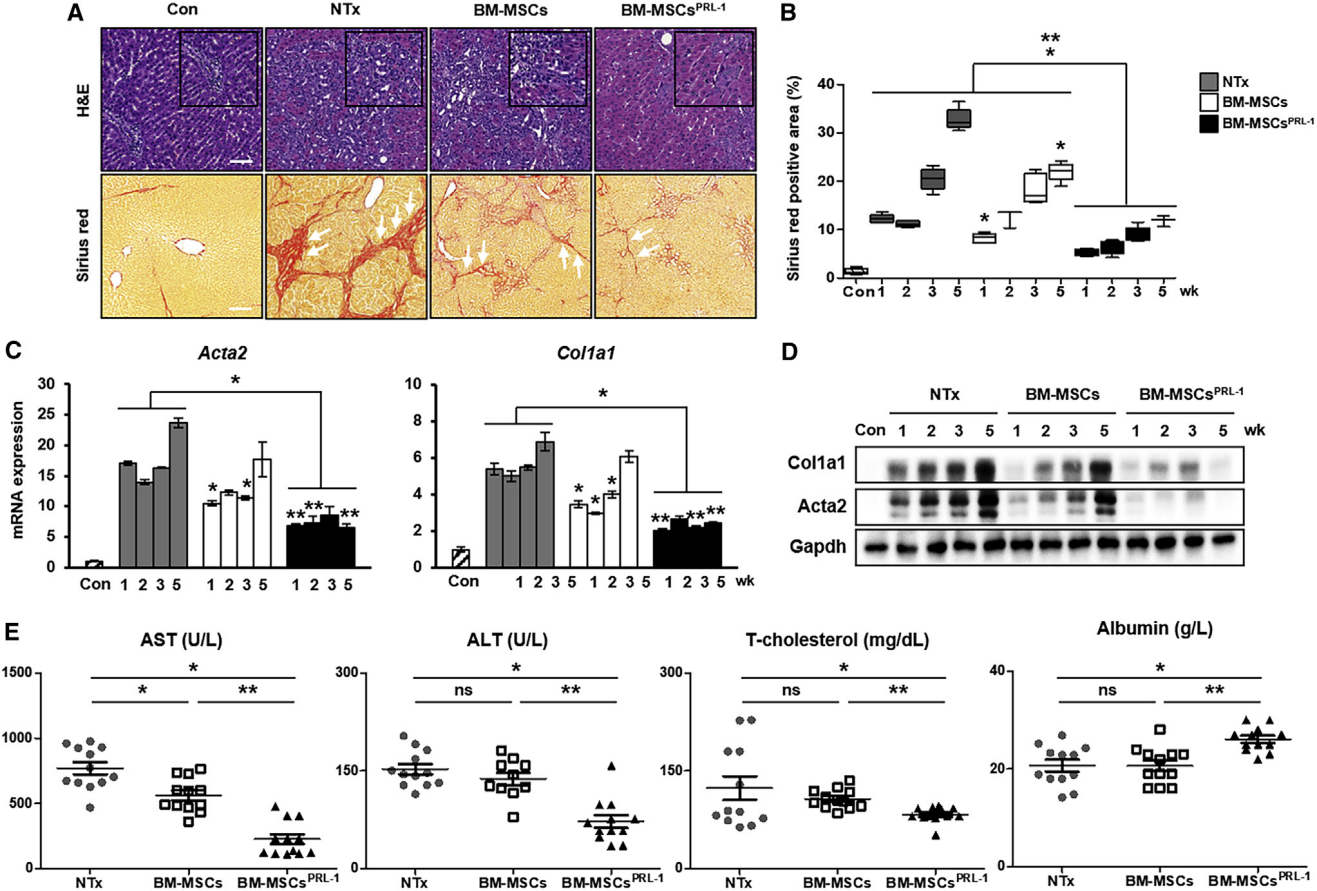
BM-MSCs^PRL−1^ had predominantly antifibrotic effects in a BDL-injured rat model (A) Representative images showing histopathological analysis of rat liver sections stained with H&E and Sirius red (arrows) after naive BM-MSC (BM-MSCs; n = 20) and BM-MSC^PRL−1^ (BM-MSCs^PRL−1^; n = 20) transplantation and without transplantation (NTx; n = 19) and in sham control (Con; n = 6) rats at 5 weeks. Scale bars, 100 μm. (B) Quantification of Sirius red-positive areas in stained liver slides at 1, 2, 3, and 5 weeks (n = 5/group). (C) qRT-PCR and (D) western blot analysis of fibrosis markers (*Acta2* and *Col1a1*) in pooled liver samples (n = 5–6/group). (E) Blood chemistry analysis to show liver function (e.g., AST, ALT, T-cholesterol, and albumin) in individual serum samples (n = 5–6/group). Values represent the mean ± SD. ∗p < 0.05 in comparison with the NTx group; ∗∗p < 0.05 in comparison with BM-MSCs.

Based on these findings, we demonstrated the efficacy of BM-MSCs^PRL−1^ generated using a nonviral system in a chronic liver disease model. Nonviral-based BM-MSCs^PRL−1^ and naive BM-MSCs were stained using PKH67 fluorescence (green) kit, and then intravenously transplanted into a BDL-injured rat model (Figure S4A). We confirmed mRNA expression of human *PRL-1* was highly increased, not *PRL-2* and *3* (Figure S4B). To analyze the hepatic fibrotic rates of BM-MSCs^PRL−1^ in the BDL rat model, histopathological analysis consisting of H&E and Sirius red staining was performed. Unlike the livers of rats in the normal control group, BDL-injured rat livers exhibited diffuse bile ductular cell proliferation and the loss of enlarged hepatocytes. Additionally, the collagen accumulation area was distinctly increased in the NTx group; furthermore, the Tx groups exhibited decreased Sirius red-stained collagen fiber areas. In particular, the Sirius red-positive area in the Tx BM-MSCs^PRL−1^ group was significantly decreased compared with that in the Tx BM-MSC group (Figures 4A and 4B; p < 0.05). Similarly, the mRNA and protein levels of actin alpha 2 (*Acta2*) and collagen type 1 alpha 1 (*Col1a1*) in the Tx BM-MSCs^PRL−1^ group were remarkably decreased (Figures 4C and 4D; p < 0.05). Additionally, we analyzed hepatic function from individual rat serum samples. Compared with the NTx group, the levels of aspartate transaminase (AST), alanine aminotransferase (ALT), total (T)-cholesterol, and triglyceride decreased in the naive BM-MSC and BM-MSCs^PRL−1^ groups. On the other hand, albumin and high-density lipoprotein (HDL) were increased in both groups. Interestingly, transplantation of BM-MSCs^PRL−1^ significantly decreased AST, ALT, and T-cholesterol, whereas increased albumin levels (Figure 4E). These data suggest that administration of BM-MSCs^PRL−1^ improved hepatic function in a rat model of BDL. These data demonstrate that BM-MSCs^PRL−1^ have exceptional antifibrotic effects and improved hepatic function in a BDL-injured rat model.

### Administration of BM-MSCs^PRL−1^ induced an antioxidant effect by mitochondrial biogenesis

**Figure 5:**
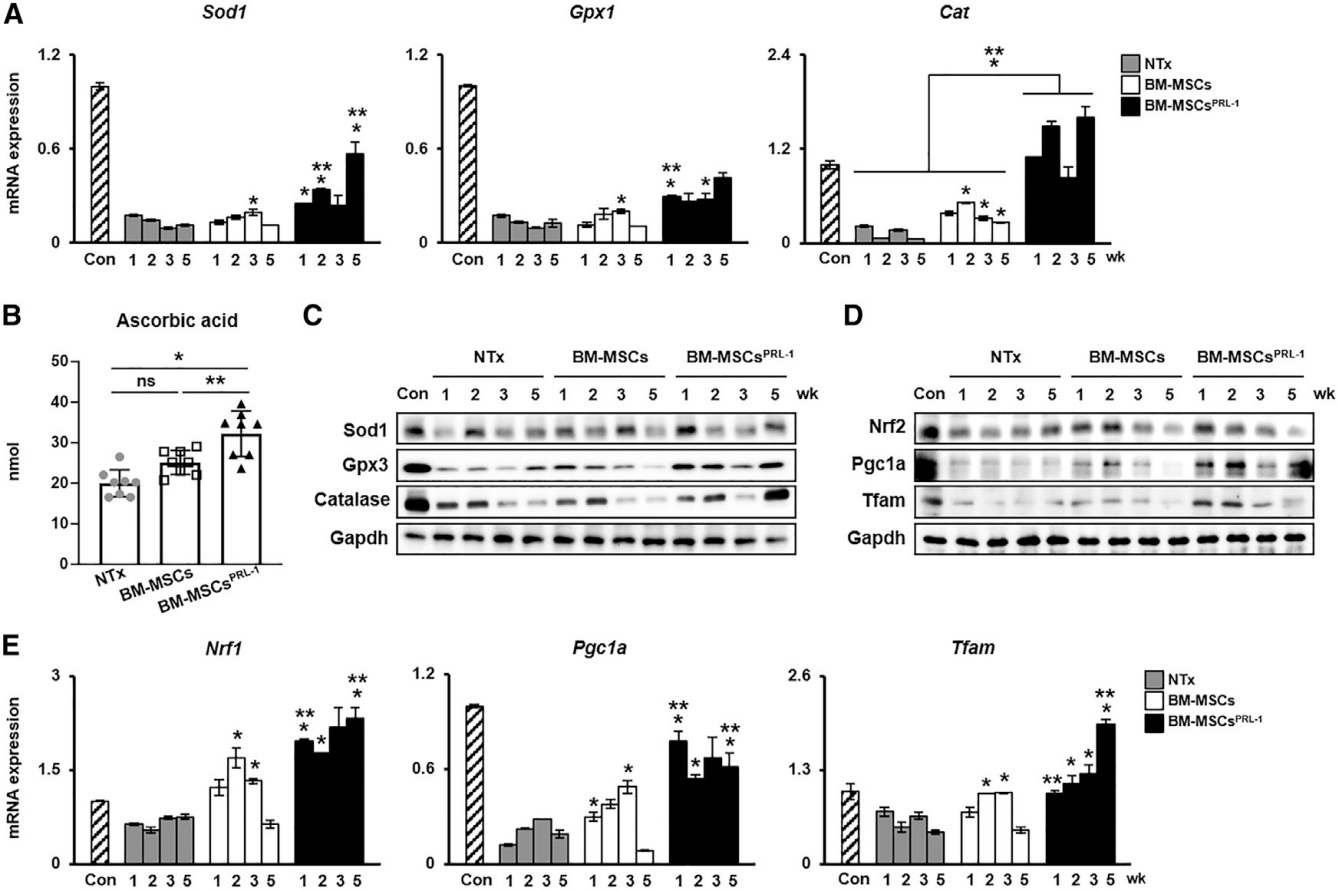
Administration of BM-MSCs^PRL−1^ had an antioxidant effect and induced mitochondrial biogenesis (A) qRT-PCR analysis of antioxidant markers in pooled liver samples (n = 5–6/group). (B) Ascorbic acid assay of individual liver proteins (n = 3–4/group). Western blot analysis of (C) antioxidant and (D) mitochondrial biogenesis markers in pooled liver samples (n = 5–6/group). (E) qRT-PCR analysis of mitochondrial dynamics markers in pooled liver samples (n = 5–6/group). Values represent the mean ± SD. ∗p < 0.05 in comparison with the NTx group. ∗∗p < 0.05 in comparison with BM-MSCs.

To demonstrate redox signaling and mitochondrial function in the BDL-injured rat model, antioxidant enzymes (e.g., *Sod1*, *Gpx1/3*, and catalase [*Cat*]) and mitochondrial biogenesis markers (e.g., *Nrf1/2*, *Pgc1a*, and *Tfam*) were confirmed after transplantation. The mRNA and protein expression of antioxidant enzymes was increased in naive BM-MSCs and the BM-MSC^PRL−1^ transplantation group compared with the NTx groups. Interestingly, BM-MSC^PRL−1^ transplantation significantly enhanced gene expression compared with that upon naive BM-MSC transplantation (Figures 5A and 5C; p < 0.05). Moreover, ascorbic acid, an antioxidant agent, was analyzed in rat liver tissue lysates from each group. Although ascorbic acid levels upon BM-MSC transplantation were no different from those in the NTx group, ascorbic acid levels were restored in the Tx BM-MSCs^PRL−1^ group to the level in Tx BM-MSCs (Figure 5B; p < 0.05). In addition, the mRNA and protein expression of mitochondrial biogenesis markers was confirmed. Compared with naive BM-MSC transplantation, BM-MSCs^PRL−1^ transplantation remarkably improved expression of these markers (Figures 5D and 5E; p < 0.05). These data suggest that BM-MSCs^PRL−1^ had antioxidant effects and regulated mitochondrial dynamics in a BDL-injured rat model.

### BM-MSCs^PRL−1^ enhanced anaerobic metabolism by improving hepatic function in the cirrhotic rat liver

**Figure 6:**
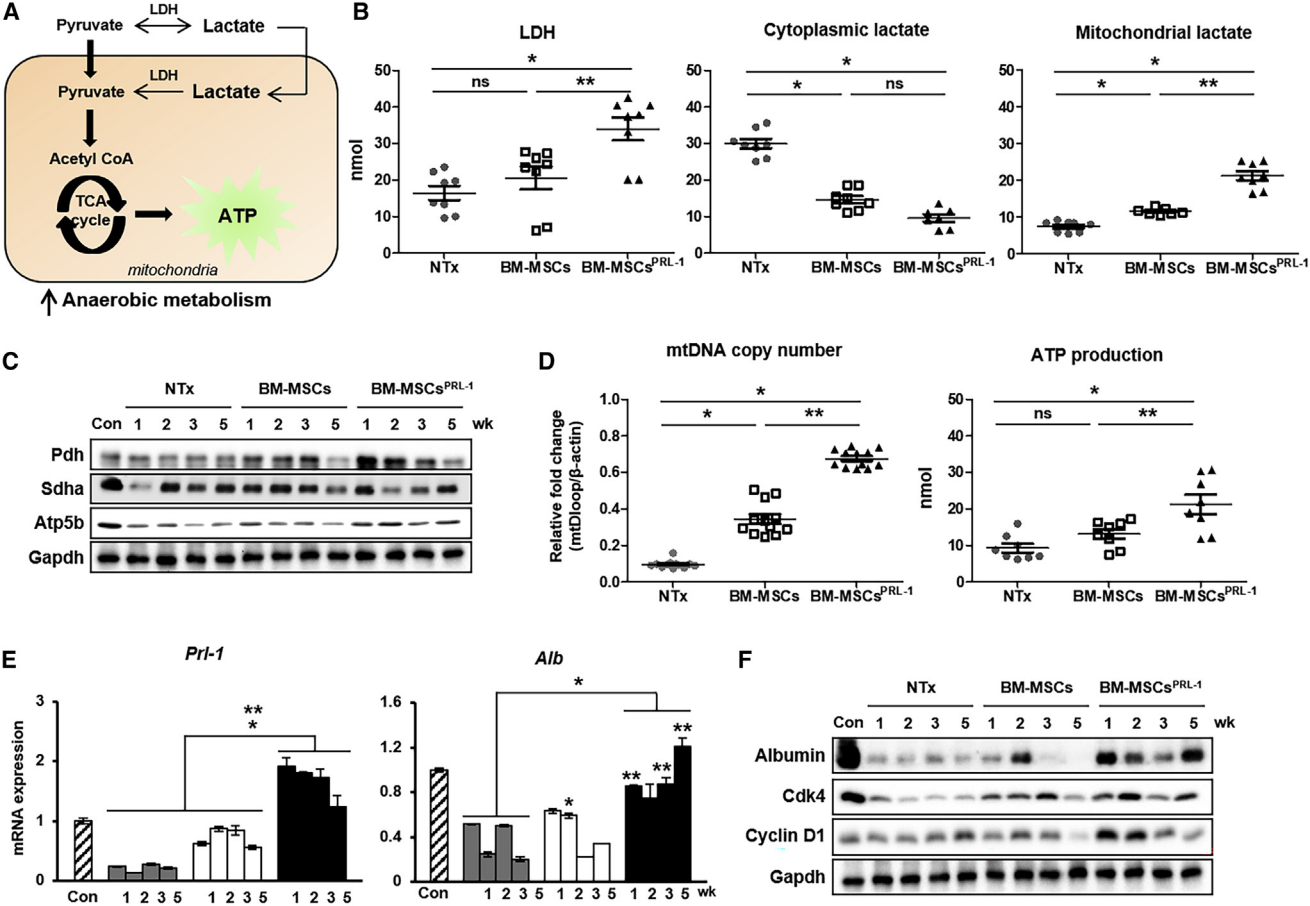
BM-MSCs^PRL-1^ enhanced anaerobic metabolism by improving hepatic function in the cirrhotic rat liver (A) Schematic diagram showing anaerobic metabolism in liver mitochondria. (B) The concentrations of LDH and cytoplasmic and mitochondrial lactate among individual liver proteins (n = 3–4/group). (C) Western blot analysis of mitochondrial respiration markers (e.g., Pdh, Sdha, and Atp5b) in pooled liver samples (n = 5–6/group). (D) mtDNA copy number and ATP production in individual liver proteins (n = 3–4/group). (E) mRNA expression of *Prl-1* and *Alb* in pooled liver samples (n = 5–6/group). (F) Protein expression of albumin and cell cycle markers (e.g., Cdk4 and Cyclin D1) in pooled liver samples (n = 5–6/group). Values represent the mean ± SD. ∗p < 0.05 in comparison with the NTx group. ∗∗p < 0.05 in comparison with BM-MSCs.

As shown in Figure 3, BM-MSCs^PRL−1^ generated using the nonviral AMAXA system provide an energy source in mitochondria and increased the rate of anaerobic glycolysis. To further demonstrate the hepatic metabolic conditions upon BM-MSC^PRL−1^ transplantation in a BDL-injured rat model, we analyzed mitochondrial metabolism through measuring the amount of lactate produced. In anaerobic glycolysis, pyruvate is converted into lactate via fermentation, and LDH catalyzes the conversion of lactate to pyruvate in a reverse reaction during the tricarboxylic acid (TCA) cycle in the liver (Figure 6A). Compared with those of the NTx group, total liver-tissue lysates of the naive BM-MSC group tended to have increased LDH levels. In the BM-MSCs^PRL−1^ group, LDH levels were significantly increased compared with those in naive BM-MSCs. To analyze lactate production in cellular organelles of the liver, total-liver lysates were fractionated into mitochondria and cytoplasm. Naive BM-MSCs showed decreased cytoplasmic lactate production compared with that of the NTx group. On the other hand, the amount of mitochondrial lactate in the naive BM-MSCs was increased compared with that in the NTx group. Interestingly, the difference in mitochondrial lactate between BM-MSCs^PRL−1^ and naive BM-MSCs was statistically significant (Figure 6B; p < 0.05). To further analyze mitochondrial function in total liver tissue, the protein expression of mitochondrial respiratory markers (e.g., Pdh, Sdha, and Atp5b) was measured and found to be increased in the Tx groups compared with the NTx group, and mtDNA copy number and ATP production were also increased in the Tx groups. In particular, mitochondrial respiratory conditions upon BM-MSC^PRL−1^ transplantation were remarkably improved compared with those upon the transplantation of naive BM-MSCs (Figures 6C and 6D). As anaerobic metabolism was enhanced by BM-MSCs^PRL−1^, the mRNA and protein expression of Prl-1, albumin, and cell cycle markers (e.g., Cdk4 and cyclin D1) was increased in BM-MSCs^PRL−1^ compared with naive BM-MSCs (Figures 6E and 6F). However, mRNA *Prl-2* and *3* expressions were not changed (Figure S5). These data suggest that the administration of BM-MSCs^PRL−1^ activated anaerobic respiration reactions and increased hepatic function in a BDL-injured rat model.

### PRL-1 modulates anaerobic metabolism through mitochondria in hepatocytes

**Figure 7:**
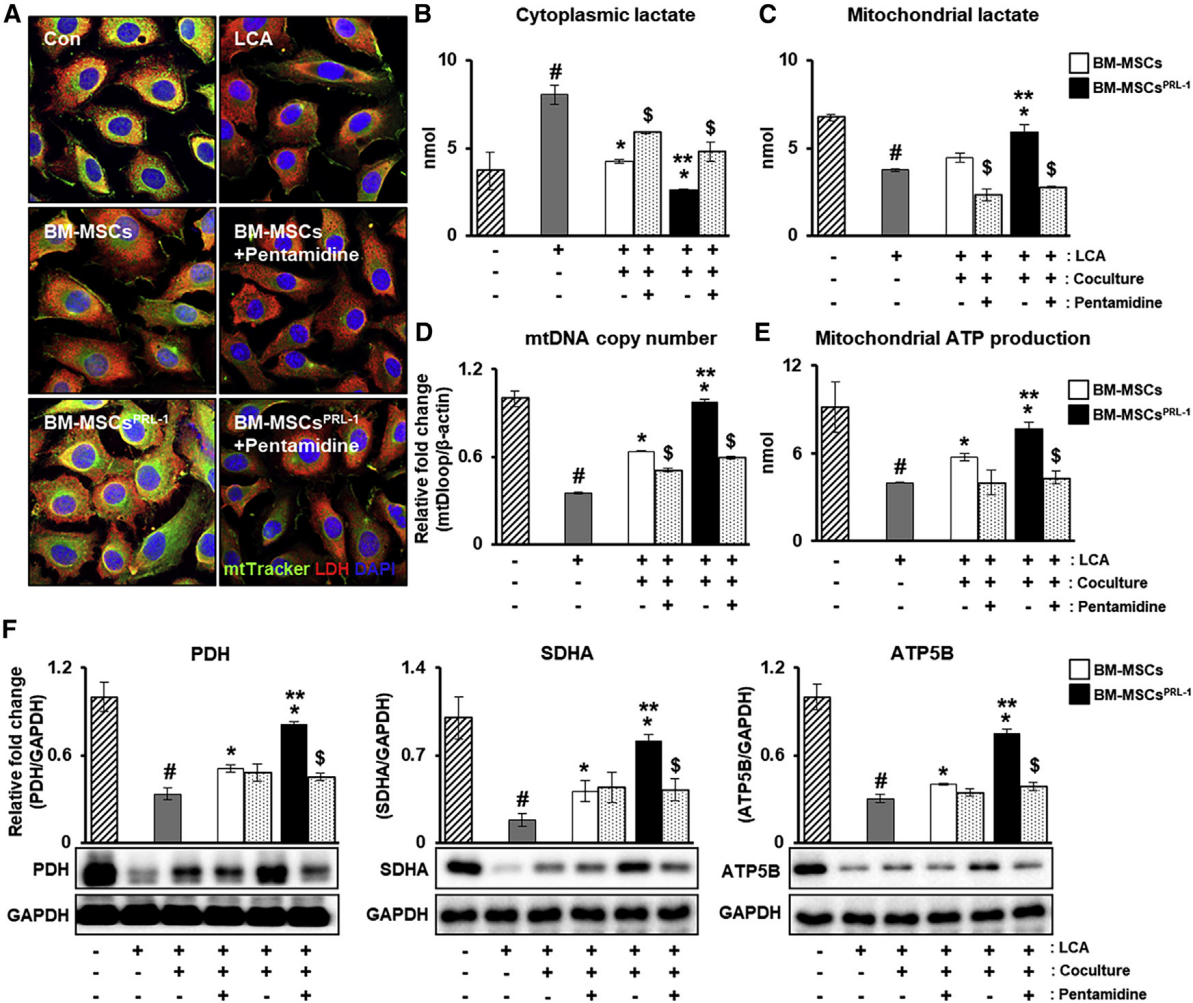
PRL-1 modulates anaerobic metabolism through mitochondria in hepatocytes (A) Immunofluorescence analysis of LDH (red) and MitoTracker (green) in LCA-injured hepatocytes cocultured with BM-MSCs^PRL−1^ upon treatment with pentamidine (1 μg/mL). The concentrations of (B) cytosolic and (C) mitochondrial lactate in hepatocytes. (D) mtDNA copy number in the gDNA determined using TaqMan assay. (E) Assay of ATP production using cell lysates (10 μg/μL). (F) Protein expression of mitochondrial respiration markers in hepatocytes. Values represent the mean ± SD. ^#^p < 0.05 in comparison with the control (−) group; ∗p < 0.05 in comparison with the LCA group; ∗∗p < 0.05 in comparison with the coculture groups; ^$^p < 0.05 in comparison with the untreated groups.

To further verify the metabolic states of the hepatocytes, treatment with lithocholic acid (LCA), a bile acid used to mimic cholestasis, was performed, and BM-MSCs^PRL−1^ were cocultured with hepatocytes with or without treatment with pentamidine, a PRL-1 inhibitor. First, immunostaining of LDH and MitoTracker staining were confirmed. Although LCA treatment decreased expression, LDH expression in mitochondria was indicated according to naive and BM-MSC^PRL−1^ cocultivation. Interestingly, BM-MSC^PRL−1^ coculture significantly increased LDH expression; however, pentamidine treatment reduced LDH expression (Figures 7A and S6). Additionally, to analyze the amount of lactate in the cytoplasm and mitochondria of the hepatocytes, fractionized samples were used. In the LCA treatment group compared with the control group, increased cytoplasmic lactate and decreased mitochondrial lactate were observed. After coculture of the hepatocytes with naive BM-MSCs and BM-MSCs^PRL−1^, lactate production was reduced in the cytoplasm but increased in mitochondria. In particular, the changes upon coculture with BM-MSCs^PRL−1^ were significantly different than those upon coculture with naive BM-MSCs. Pentamidine treatment tended to have the opposite effects (Figures 7B and 7C; p < 0.05). Additionally, mtDNA copy number and mitochondrial ATP production were remarkably improved upon BM-MSC^PRL−1^ cocultivation (Figures 7D and 7E; p < 0.05). In addition, the protein expression of mitochondrial respiratory markers was increased upon coculture with BM-MSCs^PRL−1^ compared with naive BM-MSCs. Furthermore, upon pentamidine treatment, decreased expression was observed (Figure 7F; p < 0.05). These data indicate that BM-MSCs^PRL−1^ improve anaerobic metabolism in hepatocytes.

## Discussion

Lactate is an important intermediary product in numerous metabolic processes that acts as a particularly mobile fuel for aerobic metabolism. In the liver, the net uptake of lactate is caused by gluconeogenesis, and the liver exhibits higher net lactate clearance than any other organ, with its net lactate clearance anticipated to be up to 70% of that in the whole body.^23^ The use of lactate for mitochondrial respiration requires its conversion to pyruvate by mitochondrial LDH, which fuels bioenergetics in several tissues by oxidizing lactate.^24^ Patients with acute liver failure, in contrast, have been shown to undergo splanchnic release of lactate. Hepatic lactate production in hepatic failure is thought to be due to accelerated splanchnic glycolysis, leading to the release of lactate. Lactate metabolism and organ-related lactate kinetics have not been investigated in patients with hepatic failure, but, hypothetically, circulating lactate levels would be expected to be highly related to the degree of hepatic dysfunction.^25^ However, anaerobic metabolism in a cholestatic liver model remains unknown.

Although MSC-based cell therapy has prospective potential in the treatment of hepatic failure due to the multilineage differentiation, homing, and immunomodulatory capacities of MSCs, the aging of MSCs causes an age-dependent decline in their cell number and function, resulting in limited self-renewal capacity.^26^ Aged MSCs are associated with an increase in intracellular reactive oxygen species (ROS) and MSC senescence.^27^ Therefore, to overcome the limited function of MSCs, genetic modification using gene delivery systems is an effective approach to promote the therapeutic efficacy of MSCs in liver diseases.^28^ We previously reported that *PRL-1*-overexpressing placenta-derived MSCs (PD-MSCs^PRL−1^) generated using the nonviral AMAXA system improved mitochondrial respiratory reactions at the cellular level. In addition, their transplantation in a cholestatic rat model restored hepatic function by regulating mitochondrial metabolism in the liver.^29^

In previous studies, genetic modification of MSCs using a viral-based gene delivery system has been commonly implemented as a strategy to target diseases for cell therapy because of its various advantages, such as the unique attributes of each system, providing versatility, efficiency, and ease of use.^30^^,^^31^ In several countries, autologous mesenchymal stromal cells have been drawn from eligible patients transfected with retrovirus and tested for their safe use as an investigational medicinal product (IMP) in patients in a phase 1/2 study (European Union [EU] clinical trial number 2012-003741-15).^32^ However, viral gene delivery systems typically display mutagenesis, cytotoxicity, and immunogenicity. For use in gene therapy clinical trials, these systems have various limitations. Many researchers have consistently studied nonviral vectors to prevent these elementary problems. Nonviral gene transfection is appropriate for the generation of medical products because of its safety and the lack of chromosomal integration.^33^ Recently, adipose-derived MSCs (AD-MSCs) nonvirally modified with bone morphogenetic protein 4 (*BMP4*) using polymeric nanoparticles were found to be an effective approach for the treatment of the target disease.^7^ In particular, nonviral electroporation, a known AMAXA nucleofection system, demonstrated high transfection efficiency in a population of viable MSCs using a plasmid containing the cytomegalovirus (CMV) promoter.^34^ Nucleofection has been successfully applied in primary cells and other hard-to-transfect cell lines, including MSCs, and provides unique insight into a novel experimental method as a therapeutic strategy.^35^ Our previous studies reported establishment of an optimal protocol for gene modification in PD-MSCs using nonviral nucleofection.^36^ Additionally, in a chronic liver disease model, previous studies reported that PD-MSCs^PRL−1^ generated using the nonviral AMAXA gene delivery system increased MSC engraftment into injured liver tissues and restored hepatic function.^37^

Because little is known about the capacity to characterize the enhancement of MSCs and analyze metabolic states, we further analyzed the functional enhancement of BM-MSCs^PRL−1^ generated using lentiviral or nonviral gene delivery systems (Figure 1) and found that these cells decreased MSC senescence and ROS levels and improved mitochondrial function markers (Figure 2). In the PRL-1 structure, the enzyme active site contains residue Cys-104, which engages in a disulfide bond with the nearby residue Cys-49 to affect redox regulation, resulting in redox signaling in cellular processes.^38^ Furthermore, in cellular metabolism, naive BM-MSCs displayed increased mitochondrial respiration upon the administration of oligomycin, an ATP synthase inhibitor. In contrast, BM-MSCs^PRL−1^ underwent more anaerobic glycolysis than naive GFP-expressing cells through pyruvate-to-lactate conversion and used mitochondria as their energy source (Figure 3). By regulating the amount of magnesium, PRLs are involved in mitochondrial respiration and affect ATP turnover.^22^ Among members of the PRL family, *PRL-2* was shown through its knockout (*Prl-2*^−/−^ cells) to be coupled to ATP production and decrease mitochondrial respiration to the least extent.^39^ In a BDL-injured rat model, PKH67 signals in BM-MSCs and BM-MSCs^PRL−1^ groups were confirmed. Although their expressions for rat and human *PRL-1* in rat liver tissues were increased, the expressions of *PRL-2* and *3* were not (Figures S4 and S5). In *Prl-1*-deficient mice, endogenous Prl-1 protein products were eliminated, not *PRL-2*.^40^ BM-MSC^PRL−1^ transplantation functionally ameliorated hepatic fibrosis by regulating mitochondrial and cytoplasmic lactate production and enhanced mitochondrial activity compared with that of naive BM-MSCs (Figures 4–6). *Prl-2*^−/−^ mice exhibited altered thermogenesis by the regulation of uncoupling protein-1 (Ucp1) due to changes in mitochondrial respiration.^39^ *Ucp1* expression increased levels of anaerobic glucose metabolism, upregulating pyruvate dehydrogenase kinase and LDH as well as glycolysis.^41^ Mitochondrial matrix LDH exists for pyruvate-lactate shuttling in the liver mitochondria.^8^ *Ucp1* transcription during thermogenesis and the expression pattern of *Prl-1* were assessed via RNA sequencing.^42^ In a hepatic failure rat model, Krebs cycle activity and metabolites during anaerobic glycolytic flux in mitochondria were decreased, whereas the total lactate level was increased.^43^ In addition, hepatocyte treatment with pentamidine, a PRL-1 inhibitor, inhibited mitochondrial respiration enzymes and ATP production (Figure 7). In previous reports, PRLs were overexpressed in many cancer cells and increased proliferation and migration by regulating several signal pathways, including Rho family of small GTPase, ERK1/2, and phosphoinositide 3-kinase (PI3K).^44^^,^^45^ However, the *PRL-1* gene has the major role in normal hepatic cell growth located in the cell nucleus.^18^ Its overexpression in MSCs in a cholestatic liver can promote hepatic cell growth for liver regeneration.^37^

In this study, we successfully generated BM-MSCs^PRL−1^ using lentiviral and nonviral gene delivery systems. Specifically, BM-MSCs^PRL−1^ generated using a nonviral system displayed increased anaerobic metabolism and more regulated ROS levels than the cells generated using the lentiviral system. Similarly, in a BDL-injured rat model of chronic liver disease, administration of BM-MSCs^PRL−1^ generated by a nonviral system had predominantly antifibrotic effects by enhancing anaerobic metabolism compared with that in naive BM-MSCs. We further analyzed the mechanism by which PRL-1 directly induces lactate production in mitochondria.

In conclusion, it may be beneficial to overcome the functions of naive MSCs, including their limited self-renewal ability, to augment their therapeutic effects on mitochondrial anaerobic metabolism in a cholestatic rat model. The functional enhancement of MSCs modified using a nonviral gene delivery system could provide a safe and effective approach for use in next-generation MSC-based cell therapy for degenerative diseases.

## Materials and methods

### Plasmid and lentiviral vector construction

A *PRL-1* (human protein tyrosine phosphatase type 4A, member 1 [*PTP4A1*]) plasmid was purchased (Origene, Rockville, MD, USA). The CMV6-AC vector containing the *PTP4A1* gene was digested with the restriction enzymes Sgf1 and Mlu1 and included a GFP reporter gene and neomycin. A lentiviral vector including the *PRL-1* gene was acquired from SeouLin Bioscience (Seongnam, Republic of Korea). A pLenti-RSV-EF1a vector containing *PRL-1* was constructed as a tagged protein with C-terminal GFP and puromycin. To generate a control group, we used the AMAXA pCMV-GFP vector (Lonza, Basel, Switzerland) containing kanamycin, and maxGFP was digested with the restriction enzymes Kpn1 and Bgl2.

### Gene transfection

To induce overexpression of the *PRL-1* gene, naive BM-MSCs (passage no. 7; 6 × 10^4^ cells/cm^2^) were transfected by the AMAXA system using a P1 Primary Cell 4D Nucleofector X Kit L for Human MSCs (Lonza) or a lentiviral vector system (SeouLin Bioscience). We previously assessed the nucleofection system.^36^ Cells transfected using the AMAXA system as well as pCMV maxGFP vector for control group were selected with 1.5 mg/mL neomycin and those transfected with the lentiviral system were selected with 2 μg/mL puromycin.

### Cell culture

Human BM-MSCs were acquired (Cambrex Bioscience Walkersville, Walkersville, MD, USA) and cultured in α-modified minimal essential medium (α-MEM; HyClone, Logan, UT, USA) supplemented with 10% fetal bovine serum (FBS), 4 mM L-glutamine, and 1% penicillin/streptomycin (P/S; Gibco, Carlsbad, CA, USA). We previously characterized naive BM-MSCs. The WB-F344 (rat liver epithelial cells) and WI-38 (human lung fibroblasts) cell lines were purchased from ATCC (American Type Culture Collection, Rockville, MD, USA) and cultured in α-MEM (HyClone) supplemented with 10% FBS and 1% P/S. Each cell line was maintained at 37°C in a humidified atmosphere containing 5% CO_2_.

### Cholestasis model and MSC transplantation

Seven-week-old Sprague-Dawley rats (Orient Bio, Seongnam, Republic of Korea) were purchased and housed under a 12-h light and 12-h dark cycle at 21°C –23°C under specific pathogen-free conditions. A cholestatic rat model was induced by common BDL as previously described.^29^ PKH67-labeled (green; Sigma-Aldrich, St. Louis, MO, USA) naive BM-MSCs or BM-MSCs^PRL−1^ (Tx; n = 20; 2 × 10^6^ cells) were transplanted into the tail vein. Nontransplanted (NTx; n = 20) rats were maintained as well as sham controls (Con; n = 5). After 1, 2, 3, and 5 weeks, the rats were sacrificed, and liver tissue and blood were harvested. The animal experiments had been approved by the Institutional Animal Care Use Committee (IACUC) of CHA University (Seongnam, Republic of Korea [IACUC-200033]).

### Histological analysis and blood chemistry

Liver specimens from each group (n = 5) were fixed in 10% neutral buffered formalin and embedded in paraffin. Each sample was processed at a thickness of 5 μm for H&E and Sirius red staining. Morphometric images of whole-liver sections from each group were captured and measured using a digital slide scanner (3DHISTECH, Budapest, Hungary). To analyze hepatocyte function, ALT, AST, T- cholesterol, triglyceride, and albumin were measured using serum from the blood of individual animal (DooYeol Biotech, Seoul, Republic of Korea).

### Immunofluorescence

To analyze LDH expression in hepatocytes, each cell line was fixed with 4% paraformaldehyde (PFA) for 10 min and permeabilized with 0.25% Triton X-100. Fixed cells were incubated with blocking solution (DAKO, Carpinteria, CA, USA) at room temperature for 1 h. Anti-LDH (1:200, Abcam, UK) was used as a primary antibody. An Alexa 594-conjugated antibody (1:250; Invitrogen, Carlsbad, CA, USA) was used as a secondary antibody, and MitoTracker (100 nM; Invitrogen) was applied. DAPI (Invitrogen) was used as a counterstain. Images were acquired with a confocal microscope (LSM700; Carl Zeiss, Jena, Germany).

### RT-PCR and qRT-PCR

Total RNA was extracted with TRIzol LS reagent (Invitrogen). cDNA was prepared from 500 ng/μL RNA using SuperScript III reverse transcriptase (Invitrogen) following the manufacturer’s instructions. To analyze stemness and differentiation markers in naive BM-MSCs and BM-MSCs^PRL−1^, the PCR products were electrophoresed and imaged on 2% agarose gels by staining with Gel Red (Sigma-Aldrich). To assess the levels of differentiation markers and ROS in naive BM-MSCs and BM-MSCs^PRL−1^, qRT-PCR analysis was conducted using SYBR Green PCR Master Mix (Roche, Basel, Switzerland) using a CFX Connect Real-Time System (Bio-Rad, Hercules, CA, USA). The 2-ΔΔCT method was used to determine relative mRNA expression. The data were confirmed in triplicate. PCR was carried out with specific primers (Table S1).

### Western blot analysis

To quantify specific gene expression, samples were lysed in lysis buffer (Sigma-Aldrich). Samples containing equal quantities of protein were run on 10% to 12% SDS polyacrylamide gels and transferred to polyvinylidene difluoride (PVDF) membranes, which were then blocked in 5% BSA and incubated overnight at 4°C with anti-catalase and anti-human PRL-1 (1:1,000, both from Abcam, Cambridge, UK); anti-GPX3, anti-HLAG (4h84), and anti-PGC1A (1:1,000, all from NovusBio, Littleton, CO, USA); anti-POU5F1 (1:1,000, AbFrontier, Seoul, Republic of Korea); antibodies from a Mitochondrial Marker Antibody Sampler Kit (1:1,000, Cell Signaling Technology, Danvers, MA, US); anti-HO-1 (1:500, Cell Signaling Technology); anti-ATP5B (1:500, Santa Cruz Biotechnology, Dallas, TX, USA); anti-NRF2 (1:1,000, Bioss, Woburn, MA, USA); anti-TFAM (1:1,000, Thermo Fisher Scientific, Waltham, MA, USA); and anti-GAPDH (1:3,000, AbFrontier). The membranes were then incubated with peroxidase-conjugated secondary anti-rabbit IgG and anti-mouse IgG (1:8,000, Bio-Rad). Bands were detected using enhanced chemiluminescence reagent (Bio-Rad Laboratories).

### Multilineage differentiation

#### Adipogenic and osteogenic potential

To induce adipogenic and osteogenic differentiation, fifth-passage BM-MSCs^PRL−1^ transfected using the lentiviral or AMAXA system were induced in medium from StemPro adipogenesis and osteogenesis differentiation kits for MSCs (Gibco). We changed the medium every other day. After approximately 21 days, cells transfected with each system were fixed with 4% PFA, oil red O (Sigma-Aldrich) staining of lipid was carried out to visualize lipid vesicles, and von Kossa staining was performed with 5% silver nitrate (Sigma-Aldrich) under light to evaluate the accumulation of calcium deposits.

#### Chondrogenic potential

Fifth-passage BM-MSCs^PRL−1^ using both systems were induced in medium from MesenCult-ACF chondrogenic differentiation kit for MSCs (Stem Cell Technologies, Vancouver, BC, Canada) for approximately 21 days. Each sample was fixed with 4% PFA and stained with 1% Alcian blue solution (pH 2.5; Sigma-Aldrich) to identify chondrocytes.

#### Hepatogenic potential

Fifth-passage BM-MSCs^PRL−1^ generated with both systems were induced to undergo hepatogenic differentiation as described previously. After 18 days, each BM-MSC^PRL−1^ culture was confirmed with 1 mg/mL indocyanine green (ICG; Dong in Dang Pharmaceutical, Siheung, Republic of Korea), the uptake of which was then evaluated.

#### Neurogenic potential

To induce neurogenic differentiation, fifth-passage BM-MSCs^PRL−1^ were seeded at a density of 5 × 10^3^ cells/cm^2^ using a multistep protocol. After 12 days, neurogenic differentiation was confirmed by immunofluorescence analysis of glial fibrillary acidic protein (GFAP) and nestin (NES). Cells fixed using 4% PFA were stained with polyclonal antibodies (1:200 dilution; Sigma-Aldrich) at 4°C overnight. An Alexa 488-conjugated polyclonal antibody (Invitrogen) was used as the secondary antibody, and DAPI was used for counterstaining (Vector Laboratories, Burlingame, CA, USA).

### Fluorescence-activated cell sorting analysis

To phenotype cell-surface antigens, BM-MSCs^PRL−1^ (passage no. 3) were incubated with each of the following monoclonal antibodies: anti-CD34-PE, anti-CD90-PE, anti-HLA-ABC-FITC, anti-HLA-DR-FITC (BD Bioscience, San Jose, CA, USA), anti-CD13-PE (BioLegend, San Diego, CA, USA), anti-CD105-FITC (R&D Systems, Abingdon, UK), and anti-HLAG (Abcam). The phenotype of each cell line was analyzed using a FACSCalibur flow cytometer (Becton Dickinson, San Jose, CA, USA).

### Teratoma formation

Nine-week-old male nonobese diabetic/severe combined immunodeficiency (NOD/SCID) mice (Laboratory Animal Research Center, Bundang CHA Medical Center, CHA University, Seongnam, Republic of Korea) were housed in an air-conditioned animal facility. Transfected BM-MSCs^PRL−1^ (passage no. 3) of each type (lentivirus, 5 × 10^5^/AMAXA, 5 × 10^5^) were directly transplanted into one testis (Tx; n = 2). The other testis was not injected with BM-MSCs (Con; n = 2). After maintenance for 14 weeks, the nontransplanted and transplanted testes were collected, and all mice were sacrificed. Each H&E-stained tissue section was analyzed.

### SA-β-gal staining

The senescence activity of each cell type (passage no. 3) was detected using an SA-β-gal kit (Cell Signaling) according to the manufacturer’s instructions. Cells were fixed for 10 min in a 1× fixative solution at room temperature and then incubated overnight at 37°C with a 1× SA-β-gal staining solution (pH 6.0). The percentage of SA-β-gal-positive cells among each cell type was analyzed by the program ImageJ (NIH).

### Mitochondrial DNA copy number analysis

To analyze mitochondrial DNA (mtDNA) copy number, genomic DNA was isolated from each sample (passage no. 3). Primers were targeted against mtDNA (forward, 5′-CCACTGTAAAGCTAACTTAGCATTAACC-3′; reverse, 5′- GTGATGAGGAATAGTGTAAGGAGTATGG-3′), nuclear DNA (forward, 5′-CCAGAAAATAAATCAGATGGTATGTAACA-3′; reverse,5′-TGGTTTAGGAGGGTGCTTCC-3′). The reaction mixtures contained 250 ng of gDNA template, forward primer, nuclear DNA with FAM-labeled quencher dye, reverse primer mtDNA with JOE-labeled quencher dye, and 1× TaqMan Universal Master Mix (Applied Biosystems, Foster City, CA, USA). The mtDNA copy number assay was conducted according to the manufacturer’s instructions in the 2× TaqMan Universal PCR Master Mix User Guide. All reactions were performed in triplicate.

### Mitochondrial metabolism analysis

To assess the cellular metabolic state in cells (passage no. 3) and liver tissue lysates, ATP, LDH, ascorbic acid, and lactate production rates were measured using an ATP assay kit, LDH assay kit, ascorbic acid assay kit, and L-lactate assay kit (Colorimetric; Abcam), respectively. The absorbance at 450 nm was measured using an Epoch microplate reader (BioTek, Winooski, VT, USA). Each experiment was performed in triplicate.

### Extracellular flux assay of mitochondrial stress

Real-time analysis of the ECAR, OCR, and metabolic features of naive BM-MSCs and BM-MSCs^PRL−1^ (passage no. 3) was conducted with an XF24 Extracellular Flux Analyzer (Seahorse Bioscience, North Billerica, MA, USA). Naive BM-MSCs and BM-MSCs^PRL−1^ were seeded in a 24-well XF microplate (Seahorse Bioscience) at 7 × 10^3^ cells/well the day before the analysis. The experimental techniques and program setting followed the manufacturer’s recommendation. Cells were maintained at equilibration in extracellular flux (XF) buffer in a non-CO_2_ incubator for 60 min for analyses of ECAR and OCR levels by repeated cycles of mixing (3 min), an incubation step (2 min), and measurement (3 min). For basal cellular respiration measurements, each cell type was subjected to sequential treatment with 0.5 μM oligomycin, 0.5 μM FCCP, and a 1 μM rotenone and antimycin A (AA) mixture, and changes in respiration were recorded. The OCR and ECAR were then normalized to the total cell number. All experiments were performed in triplicate.

### DT assay

To examine the DT of naive BM-MSCs and BM-MSCs^PRL−1^ (passage no. 3), approximately 5 × 10^3^ cells/cm^2^ were cultured. The DT of the harvested cells at each time point was calculated using the following formula available online (http://www.doubling-time.com): DT = t × log2/(logN1 – logN0), where *N0* is the number of cells inoculated, *N1* is the number of harvested cells, and *t* is the culture time.

### Statistical analyses

Data are expressed as the mean ± standard deviation (SD) and were analyzed using GraphPad Prism version 5.0 (GraphPad Software, San Diego, CA, USA). To compare values between two groups, two-tailed unpaired Student’s t test was performed. To compare data from multiple groups, one-way or two-way ANOVA followed by Tukey’s *post hoc* test was performed. A p value of <0.05 was used to indicate statistical significance. All experiments were conducted in duplicate or triplicate.

## Data Availability

The authors declare that all data supporting the findings of this study are available within the paper and its supplemental information.

## Supplemental information

Document S1. Figures S1–S6 and Table S1

Document S2. Article plus supplemental information
