# Supplementary material for: Increased PRL-1 in BM-derived MSCs triggers anaerobic metabolism via mitochondria in a cholestatic rat model
Source: Mol Ther Nucleic Acids. 2023 Feb 4;31:512–24. doi: 10.1016/j.omtn.2023.01.017 (PMC9970868; doi:10.1016/j.omtn.2023.01.017)
Supplement: Document S1. Figures S1–S6 and Table S1 [file mmc1.pdf]

OMTN, Volume *31*

Supplemental information

Increased PRL-1 in BM-derived MSCs triggers anaerobic metabolism via mitochondria in a cholestatic rat model

Jae Yeon Kim, Se Ho Kim, Jin Seok, Si Hyun Bae, Seong-Gyu Hwang, and Gi Jin Kim

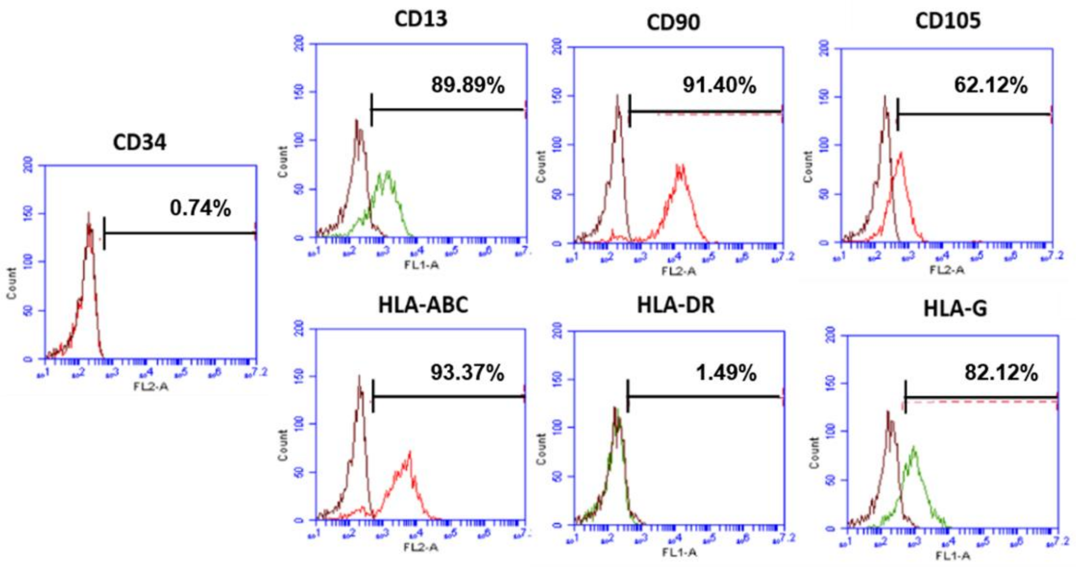

Figure S1. FACS analysis of surface markers related to hematopoietic cells and nonhematopoietic cells, and HLA family members in BM-MSCs^{PRL-1} using nonviral AMAXA gene delivery system.

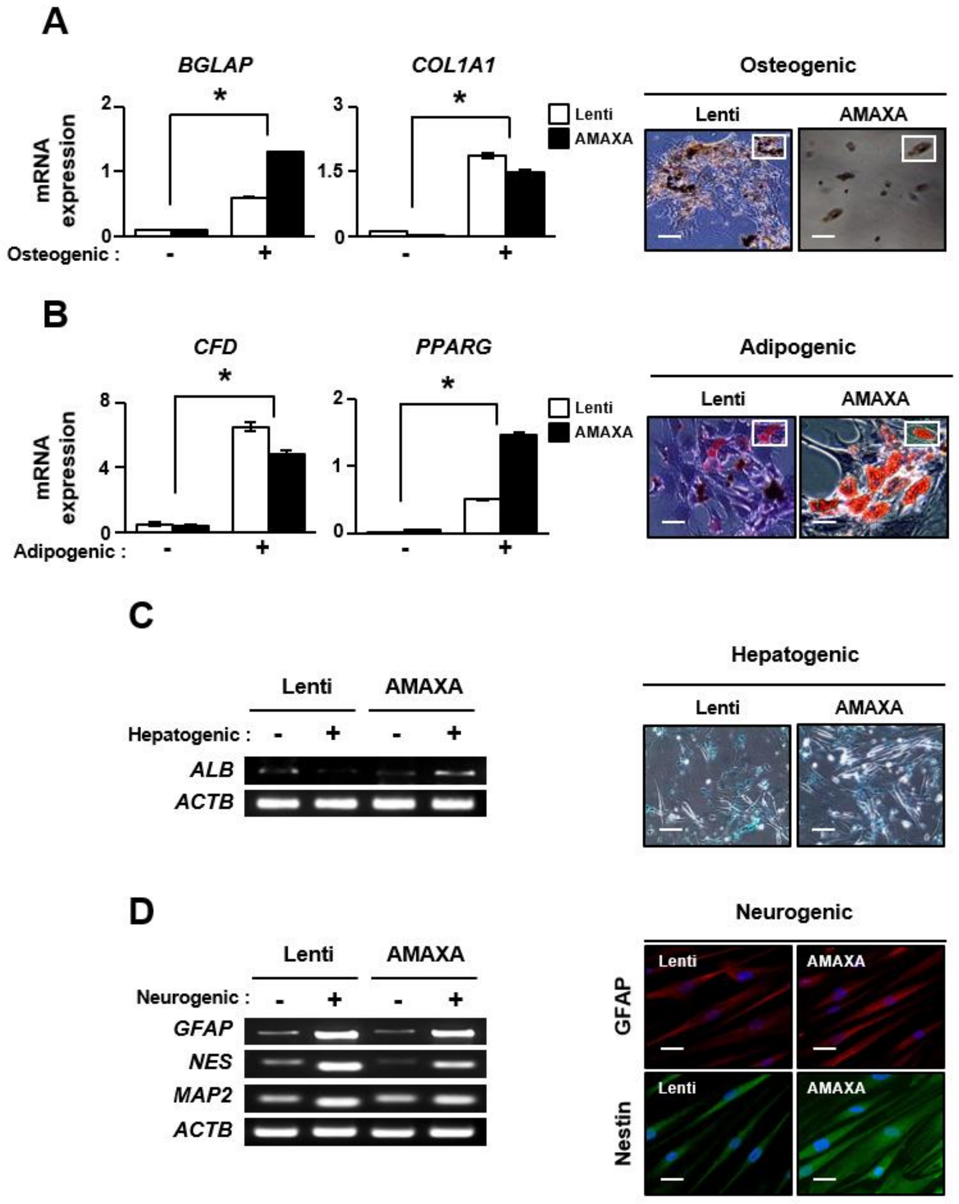

Figure S2. Potential for multi-differentiation of BM-MSCs^{PRL-1}.

(A) qRT-PCR analysis of osteogenic markers (*BGLAP* and *COL1A1*) in undifferentiated (-) and differentiated (+) BM-MSCs^{PRL-1}. Von Kossa staining for osteogenic differentiation of BM-MSCs^{PRL-1}. Scale bars = 50 μm. **(B)** qRT-PCR analysis of adipogenic markers (*CFD* and *PPARG*) in undifferentiated and differentiated BM-MSCs^{PRL-1}. Oil Red O staining for adipogenic differentiation of BM-MSCs^{PRL-1}. Scale bars = 50 μm. **(C)** RT-PCR analysis of albumin (*ALB*) expression in undifferentiated and differentiated BM-MSCs^{PRL-1}. ICG uptake

in BM-MSCs^{PRL-1} after hepatogenic differentiation. Scale bars = 50 μm.

(D) RT-PCR analysis of neurogenic markers (*GFAP*, *NES*, and *MAP2*) in undifferentiated and differentiated BM-MSCs^{PRL-1}. Immunofluorescence of GFAP (red) and Nestin (green) expression as well as DAPI (blue) BM-MSCs^{PRL-1}. Values represent the mean ± SD. $^{*}p < 0.05$ in comparison with undifferentiated groups.

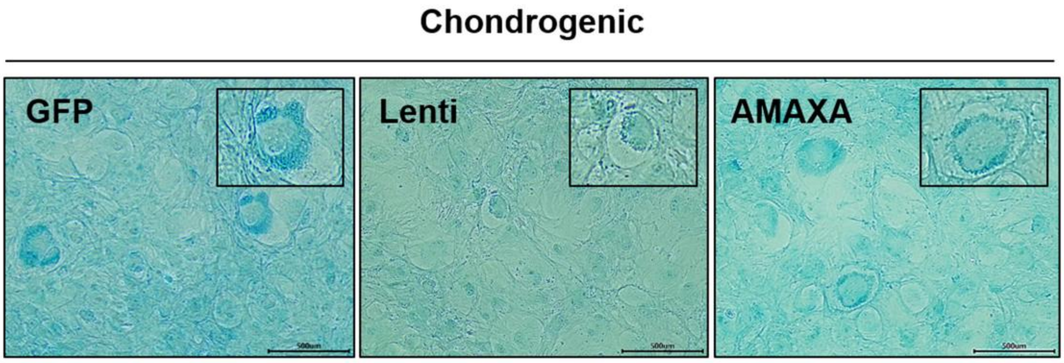

Figure S3. Potential for chondrogenic differentiation of BM-MSCs$^{PRL\text{-}1}$. Alcian staining for chondrogenic differentiation of BM-MSCs$^{PRL\text{-}1}$. Scale bars = 500 μm.

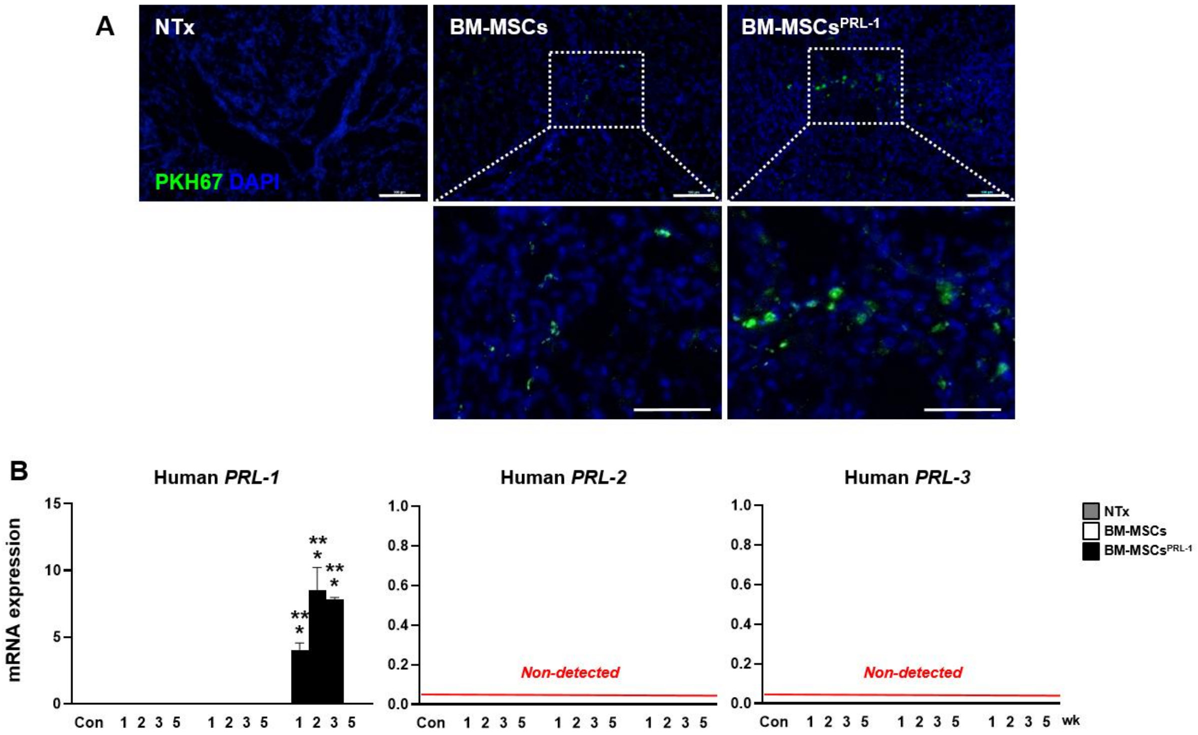

Figure S4. MSC Engraftment in BDL-injured rat liver tissues.

(A) PKH67 signals in BDL-injured rat liver tissues by fluorescence (PKH67 = green; DAPI = blue). Scale bars = 100 μm.

(B) qRT-PCR analysis of human *PRLs* in pooled liver samples (n = 5~6/group). Values represent the mean ± SD. $^{*}p < 0.05$ in comparison with the NTx group. $^{**}p < 0.05$ in comparison with BM-MSCs.

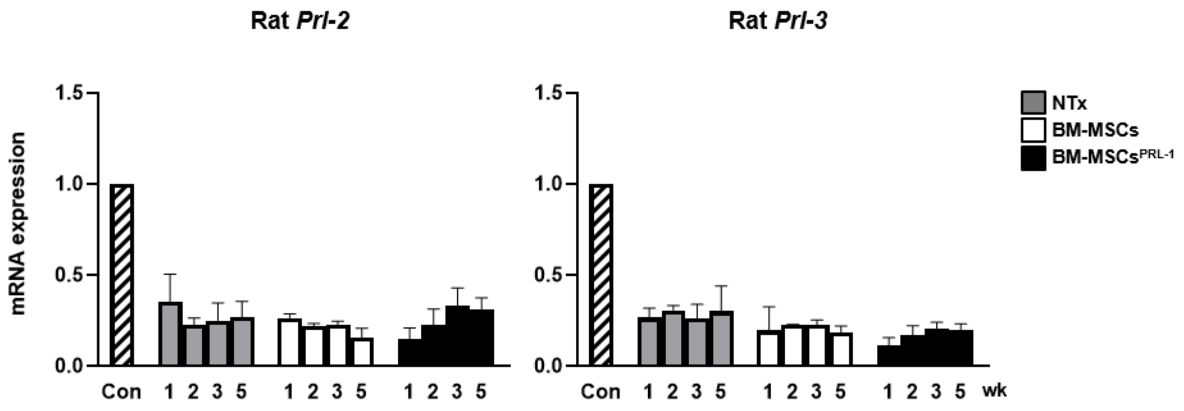

Figure S5. mRNA expression of rat PRL-2 and 3 in a BDL-injured rat liver. Values represent the mean ± SD.

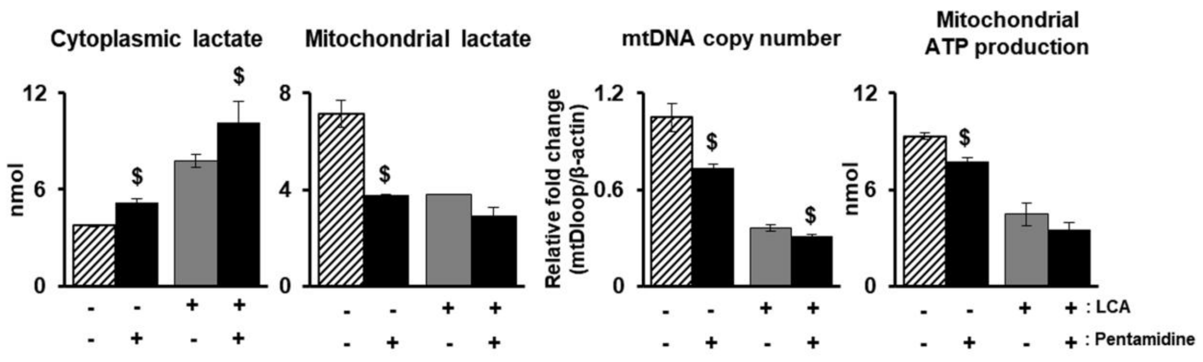

Figure S6. Cytoplasmic and mitochondrial lactate, mtDNA copy number, and mitochondrial ATP production assay in LCA-injured hepatocyte according to pentamidine (1 µg/ml) treatment. Values represent the mean ±SD. $^{\$}p < 0.05$ in comparison with non-prentamidine treatment.

Table S1: qRT-PCR primer sequences

Genes		Primer sequences	Tm
POU5F1	Forward	5'-AGTGAGAGGCAACCTGGAGA-3'	52
	Reverse	5'-GTGAAGTGAGGGCTCCCATA-3'	
NANOG	Forward	5'-TTCTTGACTGGGACCTTGTC-3'	52
	Reverse	5'-GCTTGCCTTGCTTTGAAGCA-3'	
SOX2	Forward	5'-GGGCAGCGTGTACTTATCCT-3'	52
	Reverse	5'-AGAACCCCAAGATGCACAAC-3'	
HLA-G	Forward	5'-GCGGCTACTACAACCAGAGC-3'	58
	Reverse	5'-GCACATGGCACGTGTATCTC-3'	
TERT	Forward	5'-GAGCTGACGTGGAAGATGAG-3'	55
	Reverse	5'-CTTCAAGTGCTGTCTGATTCCAATG-3'	
Albumin	Forward	5'-TGAGTTTGCAGAAGTTTCCA-3'	60
	Reverse	5'-CCTTTGCCTCAGCATAGTTT-3'	
BGLAP	Forward	5'-CACTCCTCGCCCTATTGGC-3'	58
	Reverse	5'-CCCTCCTGCTTGGACACAAAG-3'	
COL1A1	Forward	5'-AGACATCCCACCAATCACCT-3'	60
	Reverse	5'-CGTCATCGCACAACACCT-3'	
CFP	Forward	5'-GGTCACCCAAGCAACAAAGT-3'	60
	Reverse	5'-CCTCCTGCGTTCAAGTCATC-3'	
PPARG	Forward	5'-TTGACCCAGAAAGCGATTCC-3'	60
	Reverse	5'-AAAGTTGGTGGGCCAGAATG-3'	
GFAP	Forward	5'- ATCAACTCACCGCCAACA -3'	60
	Reverse	5'- CGACTCAATCTTCCTCTCCAG -3'	
NES	Forward	5'- GGTGGAGAAGGACCAAGAACTG -3'	58
	Reverse	5'- AGACCTCCTCTGTGGCATTCA -3'	
MAP2	Forward	5'- CAGGAATTGACTCCCTCTACAGC -3'	60

	Reverse	5'- TCTTCACCAGGCTTACTTTGC -3'	
HMOX1	Forward	5'- TGGTGATGGCCTCCCTGTACCACATCT -3'	60
	Reverse	5'- AGAGCTGGATGTTGAGCAGGAACGCAGTCT -3'	
HMOX2	Forward	5'- ATGTCAGCGGAAGTGGAA -3'	60
	Reverse	5'- GGGAGTTTCAGTGCTCGC -3'	
SOD1	Forward	5'- GCTGTACCAGTGCAGGTCCTCA -3'	60
	Reverse	5'- CATTTCCACCTTTGCCCAAGTC -3'	
SOD2	Forward	5'- GGAGAACCCAAAGGGGAGTTG -3'	60
	Reverse	5'- GCCGTCAGCTTCTCCTTAAAC -3'	
GPX1	Forward	5'- ACACCCAGATGAACGAGCTG -3'	60
	Reverse	5'- CAAACTGGTTGCACGGGAAG -3'	
NRF1	Forward	5'-GCTTCAGAATTGCCAACCAC-3'	60
	Reverse	5'-GTCATCTCACCTCCCTGTAAC-3'	
PGC1A	Forward	5'-CAGCAAAAGCCACAAAGACG-3'	60
	Reverse	5'-GGGTCAGAGGAAGAGATAAAGTTG-3'	
TFAM	Forward	5'-GAACAACTACCCATATTTAAAGCTCA-3'	60
	Reverse	5'-GAATCAGGAAGTTCCCTCCA-3'	
β-actin	Forward	5'-TCCTTCTGCATCCTGTCAGCA-3'	58
	Reverse	5'-CAGGAGATGGCCACTGCCGCA-3'	
